# Targeted metabolomics analysis of amino acids and acylcarnitines as risk markers for diabetes by LC–MS/MS technique

**DOI:** 10.1038/s41598-022-11970-7

**Published:** 2022-05-19

**Authors:** Shaghayegh Hosseinkhani, Babak Arjmand, Arezou Dilmaghani-Marand, Sahar Mohammadi Fateh, Hojat Dehghanbanadaki, Niloufar Najjar, Sepideh Alavi-Moghadam, Robabeh Ghodssi-Ghassemabadi, Ensieh Nasli-Esfahani, Farshad Farzadfar, Bagher Larijani, Farideh Razi

**Affiliations:** 1 Diabetes Research Center, Endocrinology and Metabolism Clinical Sciences Institute, Tehran University of Medical Sciences, Tehran, Iran; 2 Department of Clinical Biochemistry, School of Medicine, Tehran University of Medical Sciences, Tehran, Iran; 3 Cell Therapy and Regenerative Medicine Research Center, Endocrinology and Metabolism Molecular-Cellular Sciences Institute, Tehran, Iran; 4 Non-Communicable Diseases Research Center, Endocrinology and Metabolism Population Sciences Institute, Tehran University of Medical Sciences, Tehran, Iran; 5 Metabolic Disorders Research Center, Endocrinology and Metabolism Molecular-Cellular Sciences Institute, Tehran University of Medical Sciences, Tehran, Iran; 6 Metabolomics and Genomics Research Center, Endocrinology and Metabolism Molecular-Cellular Sciences Institute, Tehran University of Medical Sciences, Tehran, Iran; 7 Department of Biostatistics, School of Medical Sciences, Tarbiat Modares University, Tehran, Iran; 8 Endocrinology and Metabolism Research Center, Endocrinology and Metabolism Clinical Sciences Institute, Tehran University of Medical Sciences, Tehran, Iran

**Keywords:** Biomarkers, Diseases, Endocrinology

## Abstract

Diabetes is a common chronic disease affecting millions of people worldwide. It underlies various complications and imposes many costs on individuals and society. Discovering early diagnostic biomarkers takes excellent insight into preventive plans and the best use of interventions. Therefore, in the present study, we aimed to evaluate the association between the level of amino acids and acylcarnitines and diabetes to develop diabetes predictive models. Using the targeted LC–MS/MS technique, we analyzed fasting plasma samples of 206 cases and 206 controls that were matched by age, sex, and BMI. The association between metabolites and diabetes was evaluated using univariate and multivariate regression analysis with adjustment for systolic and diastolic blood pressure and lipid profile. To deal with multiple comparisons, factor analysis was used. Participants' average age and BMI were 61.6 years, 28.9 kg/m^2^, and 55% were female. After adjustment, Factor 3 (tyrosine, valine, leucine, methionine, tryptophan, phenylalanine), 5 (C3DC, C5, C5OH, C5:1), 6 (C14OH, C16OH, C18OH, C18:1OH), 8 (C2, C4OH, C8:1), 10 (alanine, proline) and 11 (glutamic acid, C18:2OH) were positively associated with diabetes. Inline, factor 9 (C4DC, serine, glycine, threonine) and 12 (citrulline, ornithine) showed a reverse trend. Some amino acids and acylcarnitines were found as potential risk markers for diabetes incidents that reflected the disturbances in the several metabolic pathways among the diabetic population and could be targeted to prevent, diagnose, and treat diabetes.

## Introduction

Diabetes is a prevalent chronic disease affecting millions of people worldwide associated with the development of many microvascular and macrovascular complications and leads to reduced quality of life and increased economic burden^1, 2^. Conventional risk factors and diagnostic methods of diabetes play a significant role in screening; however, there is a need for novel biomarkers that are not dependent on common clinical risk factors^3^. Diabetes does not have very apparent symptoms, especially in the early stage of the disease, and can exist many years before becoming clinically evident. In this regard, researchers have attempted to identify early diagnostic approaches for diabetes to develop strategies that could improve preventive plans and make the best use of interventions^4^.

In the last decades, it has become clear that the high-throughput approaches represent a promising avenue of research to identify biomarkers related to disease occurrence. Metabolomics is an emerging field defined as the systematic analysis of metabolites in biological systems affected by genetic and environmental features. Therefore, it is essential to discriminate the molecular fluctuations that occur in the development of diabetes to progress the health of these patients and decrease the severe consequences^5, 6^.

Several studies have been performed to assess the connection between a broad range of metabolites and diabetes progression^7^, including hexoses, amino acids, phospholipids, triglycerides, and acylcarnitine^5, 8^. However, as a result of the plentiful metabolites, different ethnicities, and study designs, outcomes vary from separate studies, and there is no comprehensive agreement concerning the application of metabolites as diabetes predictive or diagnostic biomarkers^4, 9, 10^.

Thus, we established a large case–control study to evaluate fasting plasma amino acid and acylcarnitine metabolites in diabetic patients, using targeted LC–MS/MS metabolomics.

## Results

### Baseline characteristics

The study population was composed of diabetic (n = 206) and non-diabetic (n = 206) individuals with 45–90 years age range and mostly (31%) aged 50–59 years. The majority of the participants were overweight (BMI = 25–29.9, ~ 40%) or obese (BMI ≥ 30, ~ 39%). About 55% of participants were female. Baseline characteristics are presented in Table 1. FPG, HbA1c, HDL-C, non-HDL-C, and triglyceride concentrations in the analysis groups were found to be significantly different. FPG, HbA1c, and triglyceride levels were higher in cases than in controls. HDL-C and non-HDL-C were lower in cases.

**Table 1:** Baseline characteristics of study participants.

Variable	Non-Diabetes (n = 206)	Diabetes (n = 206)	*P* value
**Gender, n (%)**			0.921
Female	113 (54.85)	113 (54.85)	
Male	93 (45.15)	93 (45.15)	
**Age (year)**	61.54 ± 11.93	61.7 ± 11.51	0.890
< 50	34 (16.51)	34 (16.51)	
50–59	64 (31.07)	64 (31.07)	
60–69	53 (25.73)	53 (25.73)	
70–79	39 (18.93)	39 (18.93)	
≥ 80	16 (7.77)	16 (7.77)	
**BMI** **(Kg/m**^**2**^**)**	28.73 ± 5.06	29.00 ± 4.9	0.582
< 18.5	1 (0.49)	1 (0.49)	
18.5–24.9	43 (20.87)	42 (20.39)	
25.0–29.9	83 (40.29)	82 (39.8)	
≥ 30	79 (38.35)	81 (39.32)	
**WC/HC (cm)**	0.94 ± 0.08	0.95 ± 0.13	0.380
**Blood pressure (mmHg)**			
Systolic	138.09 ± 21.88	140.88 ± 22.39	0.202
Diastolic	82.02 ± 12.12	82.33 ± 13.08	0.803
FPG (mg/dL)	95.1 ± 12.0	153.27 ± 62.74	** < 0.001**
HbA1c (%)	5.58 ± 0.4	7.68 ± 1.75	** < 0.001**
HDL-C (mg/dL)	42.27 ± 11.83	38.43 ± 11.42	**0.001**
Non-HDL Cholesterol (mg/dL)	100.37 ± 25.44	92.32 ± 34.32	**0.008**
Cholesterol (mg/dL)	167.92 ± 31.84	163.95 ± 43.58	0.294
Triglycerides (mg/dL)	128.98 ± 67.80	170.69 ± 157.88	** < 0.001**
Hyperlipidemia medication	16 (7.77)	53 (25.73)	** < 0.001**

Continuous variables were presented as mean ± SD, and categorical variables were presented as numbers (column percentage).

BMI: body mass index, WC/ HC: waist circumference to hip circumference ratio, FPG: fasting plasma glucose, HDL-C: high-density lipoprotein cholesterol.

Significant values are in bold.

### Metabolites with different concentrations in diabetes compared to non-diabetes

**Figure 1:**
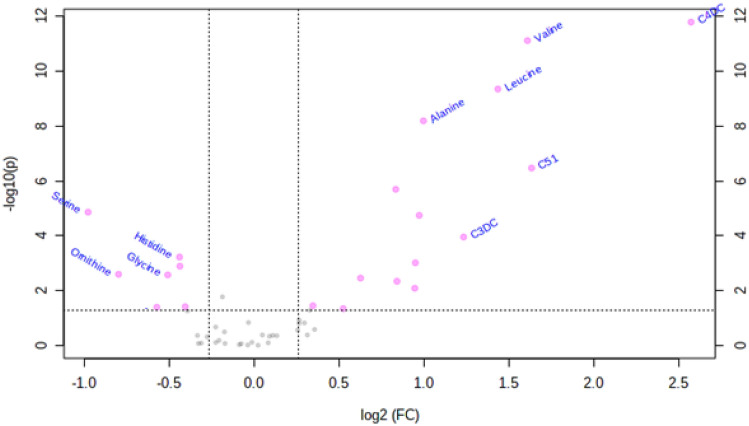
Volcano plot mainly displaying changed amino acids and acylcarnitines in diabetes compared to control group (Metaboanalyst software version 5.0).

As shown in Supplementary Table 1, many short- (C2, C3, C3DC, C4OH, C4DC, C5:1, C5OH) and long-chain (C14OH, C16OH, C18OH) acylcarnitines were increased, and only C18:1 acylcarnitine levels were decreased in diabetes compared to controls. Alanine, leucine, and valine had an increasing trend, while arginine, citrulline, glycine, ornithine, threonine, serine, and histidine had decreased significantly in diabetes. Mainly changed metabolites have been depicted by the volcano plot and represented in Fig. 1.

After adjusting for covariates (Supplementary Table 2), C3DC, C4OH, C4DC, C5:1, C5OH, C16OH, C18OH, alanine, leucine, valine, glycine, threonine, serine, and histidine alteration among study groups were independent of blood pressure and lipid profile. The effect of age, BMI, and sex, as pair-matching participants had eliminated covariates.

According to sex differences, C5, C5DC, C8:1 acylcarnitines, glutamic acid, leucine, methionine, valine, glycine, proline, and tryptophan amino acids were different between non-diabetes men and women. For diabetes, C3, C5, C5DC, C8:1 acylcarnitines along with leucine, methionine, phenylalanine, citrulline, glycine, and tryptophan amino acids were different between the two sexes (Supplementary Table 3).

### Extracted factors

Metabolite patterns were extracted using PCA with eigenvalues more than one from the scree plot (Supplementary Fig. 1). The factor-loading matrix is presented in Supplementary Table 4. As presented in Table 2, the 13 factors explained 75.5% of the total variation. The first three factors accounted for most of the overall variability; they described 22.9%, 12.5%, and 6.8%, respectively. Results of the factor analysis were acceptable as suggested by a high KMO coefficient of 0.84 and a highly significant *P* value of Bartlett sphericity test of < 0.001.

**Table 2:** Principal Component Analysis (PCA). The table lists of factors identified by PCA and the associated individual components, description, eigenvalue and variance.

Factor	Description	Components	Eigenvalue	% of Variance	Cumulative %
1	Medium-chain acylcarnitines	C5DC, C6, C8, C10, C10:1, C12, C14, C14:1, C14:2	11.438	22.876	22.876
2	Long-chain acylcarnitines	C14, C16, C16:1, C16:1OH, C18, C18:1	6.271	12.543	35.418
**3**	**BCAA and AAA**	**Tyrosine, Leucine, Valine, Methionine, Tryptophan, Phenylalanine**	**3.388**	**6.776**	**42.194**
4	Polar amino acids	Lysine, Glutamine, Asparagine, Histidine, Aspartic Acid	3.17	6.341	48.535
**5**	**Short-chain acylcarnitines**	**C3DC, C5, C5:1, C5OH**	**2.526**	**5.053**	**53.588**
**6**	**Hydroxylated long-chain acylcarnitines**	**C14OH, C16OH, C18OH, C18:1OH**	**1.888**	**3.776**	**57.364**
7	Short-chain acylcarnitines	C0, C3, C4	1.707	3.414	60.778
**8**	**Short-chain acylcarnitines**	**C2, C4OH, C8:1**	**1.529**	**3.059**	**63.837**
**9**	**Other amino acids**	**Serine, Glycine, Threonine, C4DC**	**1.378**	**2.757**	**66.594**
**10**	**Non-polar amino acids**	**Alanine, Proline**	**1.21**	**2.42**	**69.014**
11	Other amino acids	C18:2OH, Glutamic Acid	1.119	2.238	71.252
12	**Uria cycle amino acids**	**Citrulline, Ornithine**	**1.092**	**2.184**	**73.436**
13	Other amino acids	Arginine	1.012	2.023	75.459

BCAA: Branched-chain amino acids, AAA: Aromatic amino acids.

Significant values are in bold.

### Identification of the relationship between metabolite patterns and diabetes incidence

Factor 3, 5, 6, 8, 10, and 11 were positively associated with diabetes. Inline, factors 9 and 12 showed a reverse trend. Extracted factors were still associated with diabetes after adjustment for systolic and diastolic blood pressure and lipid profile, except factor 11. Results were reported in Table 3. Contrary to our expectation, the association between the first two extracted factors and diabetes incidence was insignificant.

**Table 3:** Crude and adjusted odds ratios (OR) and their 95% confidence intervals (CI) of the extracted factors analyzed the relationship between metabolite patterns and diabetes incidence.

Factor	Model 1			Model 2			Model 3		
	OR	95% CI	*P* value	OR	95% CI	*P* value^†^	OR	95% CI	*P* value^††^
1	0.997	(0.964–1.03)	0.875	0.996	(0.962–1.03)	0.814	1.003	(0.968–1.04)	0.871
2	0.973	(0.916–1.03)	0.366	0.967	(0.91–1.03)	0.286	0.971	(0.91–1.036)	0.377
3	1.096	(1.03–1.17)	**0.005**	1.1	(1.0311.17)	**0.004**	1.069	(1.02–1.14)	**0.047**
4	0.968	(0.909–1.03)	0.322	0.968	(0.908–1.03)	0.312	0.978	(0.915–1.05)	0.507
5	1.284	(1.15–1.43)	** < 0.001**	1.277	(1.15–1.42)	** < 0.001**	1.275	(1.14–1.42)	**0.001**
6	1.17	(1.06–1.29)	** < 0.001**	1.162	(1.05–1.29)	** < 0.001**	1.164	(1.05–1.29)	**0.005**
7	1.064	(0.93–1.22)	0.367	1.063	(0.929–1.22)	0.373	0.992	(0.857–1.15)	0.91
8	1.237	(1.09–1.41)	** < 0.001**	1.231	(1.08–1.40)	**0.002**	1.243	(1.09–1.43)	**0.002**
9	0.625	(0.537–0.73)	** < 0.001**	0.617	(0.529–0.72)	** < 0.001**	0.618	(0.526–0.727)	** < 0.001**
10	1.457	(1.22–1.74)	** < 0.001**	1.46	(1.22–1.74)	** < 0.001**	1.335	(1.11–1.61)	**0.002**
11	1.292	(1.05–1.58)	**0.014**	1.297	(1.058–1.59)	**0.012**	1.182	(0.962–1.45)	0.112
12	0.767	(0.647–0.908)	** < 0.001**	0.767	(0.647–0.91)	**0.002**	0.795	(0.666–0.949)	**0.011**
13	0.793	(0.626–1.01)	0.056	0.8	(0.631–1.01)	0.065	0.783	(0.611–1.00)	0.058

*P* value, crude model (Model 1).

*P* value^†^, adjusted by blood pressure (Model 2).

*P* value^††^, adjusted by blood pressure and lipid profile (HDL-C, cholesterol, triglyceride) (Model 3).

Significant values are in bold.

### Pathway analysis

**Figure 2:**
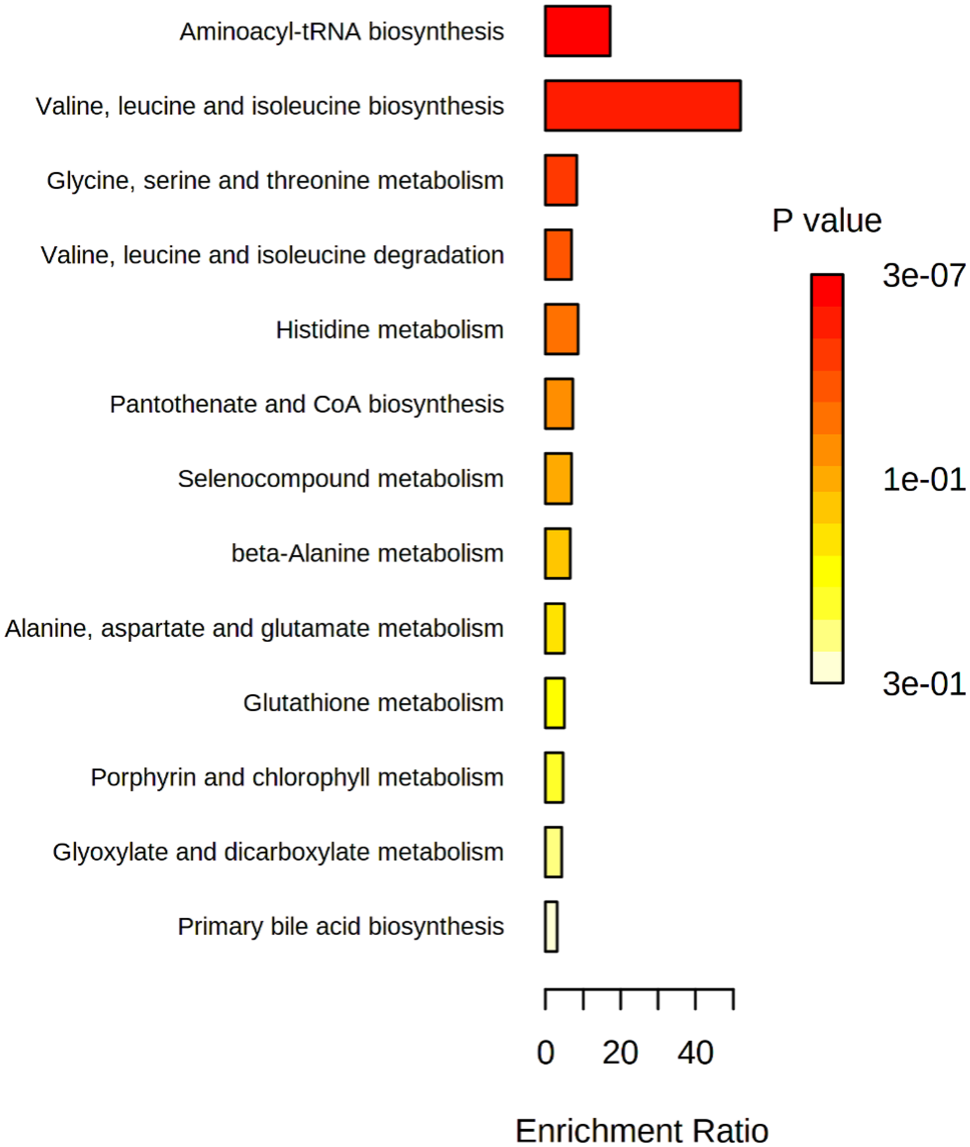
Pathway analysis. Enrichment overview (Metaboanalyst software version 5.0).

Based on differential metabolites, the disturbed pathways were determined and shown in Fig. 2, and a detailed pathway analysis table is presented in Supplementary Table 5.

For diabetes versus non-diabetes, valine, leucine, and isoleucine biosynthesis had the most enrichment ratio. Enrichment Ratio is computed by Hits/Expected, where Hits, refer to observed hits, and Expected, refers to expected hits. After that, aminoacyl-tRNA biosynthesis, serine, glycine and threonine metabolism, leucine, isoleucine, and valine degradation had the most enrichment ratio.

## Discussion

An emerging assumption is that the combinations of known biomarkers in a further personalized approach are required to give more effectiveness in terms of evaluating the degree or progress of diabetes and prove the most reliable preventive or diagnostic plans^11, 12^.

By a short review of the published articles about metabolomics analysis (comprising amino acids or acylcarnitines) in blood samples of diabetic patients in the PubMed database (Table 4), we found that studies mainly had cohort design, and most of the others had used part of cohort data and designed as nested case–control or cross-sectional. In most of them, the number of diabetic patients was close to 200–300 people but the number of controls varied within studies. Some were matched 1:1, such as our study and some others had no limitation in the number of controls.

**Table 4:** Literature review on biomarkers in diabetes metabolomics studies.

Refs.	First author, year	Study design	Ethnicity/Country	Study population		Average age		Average BMI		Metabolites		
				Controls (M/F)	Patients (M/F)	Controls	Patients	Controls	Patients	Amino acid	Acylcarnitine	Other metabolites
^44^	Kwang Seob Lee, 2021	Nested case–control	Korea	500 (210/290)	204 (85/119)	54.0 (47.0–61.0)**	57.0 (50.5–63.5) **	26.7 (25.6–27.8)**	27.25 (25.75–28.75)**	Yes	Yes	Yes
^45^	Yishuang Duan, 2021	Case–control	China	60 (33/27)	60 (33/27)	56 ± 7	56 ± 7	25.0 ± 2.7	25.0 ± 2.7	Yes	No	Yes
^46^	Lichao Wang, 2020	Cohort (3 cohorts)	China	Set-1 76 (40/36) Set-2 64 (29/35) Set-3 40 (18/22)	174 (82/92) 108 (52/56) 77 (38/39)	49.09 ± 13.08 41.20 ± 16.29 40.50 ± 16.16	50.70 ± 10.75 52.81 ± 11.80 51.17 ± 11.95	23.97 ± 3.53 22.97 ± 4.25 23.89 ± 3.66	24.81 ± 3.58 25.18 ± 3.24 24.93 ± 3.74	Yes	Yes	Yes
^14^	Samuel H Gunther, 2020	Prospective Study	Singapore	2999 (1366/1633)	314 (152/162)	47.0 ± 11.6	53.5 ± 11.2	23.1 ± 3.8	26.3 ± 4.8	Yes	Yes	No
^8^	Xin Li, 2020	Cohort	China	54 (38/16)	Simple diabetes 21 (10/11)	54.22 ± 13.33	50.82 ± 9.73	22.86 ± 3.21	25.15 ± 2.53	Yes	Yes	No
					Diabetic complication 103 (60/43)		55.78 ± 9.23		24.35 ± 2.69			
^47^	Jumana Y Al-Aama, 2019	Case–control	Saudi Arabia (middle east population)	33	34	37.65	52.94	–	–	Yes	No	Yes
^48^	Marta Guasch-Ferre, 2019	Case-cohort	Mediterranean population	641 (233/408)	251 (113/138)	66.5 ± 5.7	66.4 ± 5.7	29.7 ± 3.5	30.8 ± 3.4	No	Yes	No
^49^	Yonghai Lu, 2019	Nested case–control	China	Prevalent diabetes 144 (62/82)	144 (62/82)	62.7 ± 5.9	62.7 ± 6.1	23.1 ± 3.3	24.6 ± 3.6	Yes	No	No
				Incident diabetes 160 (79/81)	160 (79/81)	61.9 ± 6.0	61.6 ± 5.6	22.6 ± 3.5	24.6 ± 3.4			
^50^	Casey M. Rebholz, 2018	Subset of a cohort	USA	1813 (732/1081)	1126 (479/674)	53.6 ± 5.8	52.8 ± 5.5	27.2 ± 5.1	30.0 ± 6.0	Yes	No	Yes
^51^	Jordi Merino, 2018	Prospective study	USA	1055 (419/636)	95 (52/43)	53 ± 10	54 ± 9	26.18 ± 4.28	29.66 ± 5.18	Yes	No	Yes
^12^	Lin Shi, 2018	Nested case–control	Swedish population	503 (224/279)	503 (224/279)	50.1 ± 8.0	50.2 ± 7.9	25.5 ± 3.8	29.5 ± 4.9	Yes	No	Yes
^52^	Gopal Peddinti, 2017	Nested case–control	Finland	397 (200/197)	146 (74/72)	48.22 ± 0.72*	52.34 ± 0.99*	25.91 ± 0.19*	28.46 ± 0.37*	Yes	No	Yes
^53^	Jun Liu, 2017	Cohort	Netherlands	2564 (1132/1432)	212 (108/104)	48.2 ± 14.3	59.8 ± 11.8	26.7 ± 4.6	30.0 ± 5.9	Yes	No	Yes
				1434 (595/839)	137 (78/59)	47.7 ± 13.9	57 ± 10.7	26.6 ± 4.4	30.1 ± 5.1			
^54^	Birgit Knebel, 2016	Cohort	Germany	129 (46/83)	T1D 127 (79/48)	58 ± 11	35 ± 13	26.3 ± 4.4	24.6 ± 4.3	Yes	Yes	Yes
					T2D 244 (155/89)		53 ± 11		31.7 ± 5.9			
^55^	Gaokun Qiu, 2016	Nested case–control ^2cohorts^	China	1039 (464/575)	1039 (464/575)	62.93 ± 7.32	62.82 ± 7.23	23.64 ± 3.07	25.73 ± 3.34	Yes	Yes	Yes
				520 (181/339)	520 (181/339)	53.74 ± 10.18	53.82 ± 10.25	23.70 ± 3.22	25.53 ± 3.42			
^56^	Yonghai Lu, 2016	Nested case–control	Chinese men and women in Singapore	197 (80/117)	197 (80/117)	55.1 ± 2.7	55.2 ± 2.9	22.7 ± 3.1	25.5 ± 3.8	Yes	Yes	Yes
^20^	Therese Tillin, 2015	cross-sectional	European and South Asian men	2286 (2286/-)	1444 (1444/-)	51.75 ± 7.15	51.6 ± 7.1	25.56 (23.59–27.75)**	25.46 (23.5–27.5)**	Yes	No	No
^28^	Anna Floegel, 2013	Case-cohort	Germany	2282 (867/1415)	800 (462/338)	49.5 ± 8.9*	54.7 ± 7.3*	26.1 ± 0.09*	30.1 ± 0.15*	Yes	Yes	Yes
^29^	Cristina Menni, 2013	Cross-sectional	U. K	1897 (-/1897)	115 (-/115)	50.02 ± 14.43	63.00 ± 9.61	25.42 ± 4.55	30.58 ± 6.32	Yes	No	Yes

**Data reported as median (IQR); *Data reported as mean ± SEM; others reported as mean ± SD.

M: Male; F: Female; BMI: Body mass index.

Regarding ethnicity, a few publications are available on the middle east population. We could not find another metabolomics study in blood samples of Iranian or middle east populations of diabetes. As reviewed by Sonia Liggi et al. in 2017, there was no cohort study assessing metabolites that have been conducted on the middle east population until that time^13^.

For average age and BMI, most of the studies were age-matched or had study groups in a similar range of age and were mostly conducted in 40–60 decades of life. Diabetic participants usually had higher BMI than controls and took part in the obese category.

Evaluated metabolites varied, but the number of studies on acylcarnitines was small.

In this regard, we investigated the metabolic perturbations of amino acids and acylcarnitines among diabetes and non-diabetic individuals.

By factor analysis, 13 factors were extracted by PCA method, each of them mostly comprised of chemically and functionally correlated metabolites (Table 2). Factors 1 (medium-chain acylcarnitines), factor 2 (long-chain acylcarnitines), factor 4 (polar uncharged (glutamine and asparagine), positively charged (histidine and lysine), and negatively charged (aspartic acid) amino acids), factor 7 (C0, C3, C4 acylcarnitines), factor 11 (C18:2OH and glutamic acid), and factor 13 (arginine) were not significant after adjustment by blood pressure and lipid profile. Factor 3 (Branched-chain amino acids (BCAA) and aromatic amino acids (AAA), factor 5 and 8 (short-chain acylcarnitines), factor 6 (hydroxylated long-chain acylcarnitines), factor 9 and 10 (other amino acids), and factor 12 (Urea cycle amino acids) were associated with the diabetes incidence independent of confounders and have been discussed.

### Amino acids

#### Branched-chain amino acids (BCAA) and aromatic amino acids (AAA)

As the main finding of this study, Factor 3 comprised of tyrosine, valine, leucine, methionine, tryptophan, and phenylalanine was positively associated with diabetes which is consistent with other findings that have previously been published.

According to the earlier studies that evaluating the relation between metabolites and type 2 diabetes (T2D) using high-throughput metabolomics techniques, a sum of or individual BCAAs comprised of isoleucine, leucine, valine, and AAA, including tyrosine, phenylalanine, and tryptophan, were related to a higher risk of T2D^8, 10, 12, 14–16, 32^. Previous reports have associated the AAAs with an escalated risk of developing type 1 diabetes (T1D). Additional investigations have demonstrated that BCAAs elevate insulin action and signaling procedures, while others have reported that they get worse insulin resistance in individuals with T1D^1^. Nevertheless, further investigations are needed to elaborate that these amino acid levels are only markers of diabetes or be part of the cause of insulin resistance and etiology of diabetes^16^, particularly in T1D.

As stated by Libert et al.^15^, these analytes and ratios increase significantly in T2D and obesity as though about 80% of our study participants were overweight or obese. Observational studies have revealed BMI to be associated with the risk of diabetes^17^. In this respect, significant alterations in BMI and HbA1c between different ethnic groups have been found^18^. Furthermore, analyses have identified the differences in AAA and BCAA concentrations between the different ethnic groups^14, 19, 20^ and suggest the requirement for multiethnic studies to consider these ethnic differences, especially in the Middle East.

Any other way, BCAAs and AAAs (except tyrosine) are essential and dependent on diet. Interestingly, these amino acid patterns are related to the overall dietary pattern rather than the dietary intake^21^. A suggested biological mechanism concerning increased BCAAs concentration along with insulin resistance and diabetes is an inadequate response of muscle tissue to the anti-catabolic function of insulin. Subsequently, proteolysis of skeletal muscle increases and causes increased BCAAs concentration as the dominant amino acids in this tissue. In another explanation, during a series of enzymatic reactions, BCAAs metabolize to branched-chain keto acids and then oxidize to be used as substrates in the tricarboxylic acid (TCA) cycle. High BCAAs may arise from the defect of these reactions and the accumulation of intermediate compounds involved in diabetes development^22^.

For the AAAs, phenylalanine is converted into tyrosine in its catabolic pathway. Accordingly, alterations in one of them will possibly affect the other one. It has been suggested that increased levels of phenylalanine and tyrosine may result from decreased tyrosine aminotransferase activity that converts tyrosine to other intermediary compounds^22^. It has been proposed that tyrosine can inhibit insulin signaling pathways and glucose transport^23^.

#### Urea cycle amino acids

More specifically, citrulline and ornithine as components of Factor 12 were reversely associated with the incidence of diabetes. The trend of ornithine in diabetes development was in line with Cao et al.^24^, which can be referred to as inflammatory conditions. Meanwhile, it can stem from increased depletion of ornithine as a precursor for proline^25^. Contrary to these findings, in Gunther SH et al.’s study^16^, the ornithine was positively associated with the risk of diabetes which can be resulted from upregulated arginase activity, an enzyme that takes part in ornithine production.

#### Other amino acids

We also confirmed several previously reported associations of diabetes and some amino acids, including in Factor 10 (alanine, proline) and Factor 9 (C4DC, serine, glycine, threonine). Taken together, our findings support other reports as higher concentrations of proline and alanine were detected in diabetes than non-diabetes which were associated with a higher risk of T2D. The levels of glycine and serine were lower in diabetes^4, 9, 14, 16, 26^.

In this class, glycine has the most robust involvement in the pathophysiology of diabetes. Glycine is mainly synthesized from threonine and serine. It is an insulin secretagogue; therefore, a low glycine concentration may decline pancreatic insulin secretion. This decrease of glycine may be due to the increased glycine consumption for the generation of glutathione or related to its role as a neurotransmitter, increased absorption of glycine in insulin-resistant tissues for gluconeogenesis, or the opposite link between glycine and visceral-subcutaneous adipose tissue mass^22, 27, 28^.

#### Acylcarnitines

We perceived three factors that were positively associated with diabetes as follows: Factor 5 (C3DC, C5, C5OH, C5:1), factor 8 (C2, C4OH, C8:1), which is mainly composed of short-chain acylcarnitines, and factor 6 (C14OH, C16OH, C18OH, C18:1OH) contains hydroxylated long- chains acylcarnitines.

Acylcarnitines are a form of fatty acids needed to transport into mitochondria and peroxisomes for beta-oxidation, and changes in their abundance may point to a deficiency in fatty acid oxidation, glycolysis, and BCAA metabolism. It is conceivable that the accumulation of these pathways intermediates in the blood may play a key role in insulin action and progression of diabetes^29, 30^.

#### Short-chain acylcarnitines

Our study confirmed that levels of various acylcarnitines in patients with T2D had been increased^26, 31^. An increasing trend of short-chain acylcarnitines has been detected in obesity and T2D^15^. In a cohort study, the baseline C3DC, C5, C5OH, and C8:1 were related to a higher risk of T2D^32^. Notably, the short-chain acylcarnitines C3, C4-DC, and C5 derive from different energy sources as well as the BCAAs^29^. Also, C3-DC is another elevated amino acid—derived acylcarnitine that emphasizes evaluating the association between amino acids and acylcarnitines in disease development^33^. In contrast to the presented results, Muilwijk et al. demonstrated a negative association between most acylcarnitines especially short-change ones and the risk for T2D^10^.

#### Hydroxylated long-chain acylcarnitines

Long-chain acylcarnitines are going above when fatty acid supply surpasses the capacity or demand of mitochondrial oxidation and the TCA cycle^29, 34^. Additionally, acylcarnitine C16OH was significantly linked to higher diabetes risk^14^. Similar results were reported about baseline concentrations of C14OH and C18OH^32^. The remarkable thing about acylcarnitines is that although changes in these metabolites have been studied extensively, in different studies, different types of metabolites reported as significantly changed, which makes it challenging to reach a comprehensive conclusion. Confirms the need for further studies.

#### Differences according to sex

Sex as an endogenous factor can influence the human metabolome and provide valuable information through precision medicine^35^. According to sex differences, C5, C5DC and, C8:1 acylcarnitines and leucine, methionine, glycine and, tryptophane amino acids were different between men and women in both study groups. All the mentioned metabolites had higher levels among men, except C8:1 and glycine. These findings were in line with Kirstin Mittel strass et al.'s results^36^. Regardless of gender differences, just leucine and glycine significantly differed between diabetes and non-diabetes.

In Burcu F. Darst et al.'s study on healthy participants, C5, methionine, tryptophan, glutamate, proline, phenylalanine, and the BCAAs associated with sex and were in higher levels in men, while glycine was found in a lower level in men^37^. In the following in our study, higher levels of glutamic acid valine and proline amino acids were found in non-diabetic men. For diabetes, higher levels of C3 acylcarnitine along with phenylalanine and citrulline amino acids were observed in men. Just C3, citrulline, and valine changes among diabetes and non-diabetes were significant without consideration of sex.

Consistent with our findings, other studies have confirmed amino acids, particularly BCAAs, as sex-dependent metabolites, and higher concentrations of several amino acids have been reported in men, which can be caused by higher protein intake or larger muscle mass^38–40^.

## Conclusion

This study was performed on participants of a multi-regional cohort in Iran. Participants with diabetes were randomly selected, and the control group was matched with them to improve study efficiency and statistical precision. Along with amino acids, acylcarnitines, as less studied metabolites compared to others, were analyzed to be able to add new data to existing findings.

BCAAs, AAAs, and a wide range of amino acids along with many short- and long-chain acylcarnitines have been strongly associated with the risk of diabetes incidence. Our results displayed that the predictive factors contained specific amino acids and acylcarnitines would be helpful to reflect the metabolic pathway disturbances among diabetes. They might be advantageous for evolving the preventive, diagnostic, and therapeutic strategies for diabetes and its complications. In this field of study, several articles have been published in the population of countries with different races, ethnicities, and environmental conditions. However, according to our knowledge, no study has examined the status of metabolites in the population of diabetics in Iran so far. Although, further multiethnic extensive studies with the valuation of dietary patterns and assessment of probable complications are required to obtain more conclusive results.

## Material and methods

### Patients and experiments design

A case–control study was set up with 412 subjects (206 non-diabetic and 206 diabetic individuals) that were randomly chosen from participants of the Surveillance of Risk Factors of NCDs in Iran Study (STEPS 2016). STEPS is conducted periodically in 30 provinces of Iran to investigate none communicable risk factors^41^. Gender, age, waist circumference, hip circumference, body mass index (BMI), systolic and diastolic pressure were documented. Biochemical characteristics of patients, including; HbA1c, fasting plasma glucose (FPG), and lipid levels (HDL-C, cholesterol, triglyceride), were measured by Cobas C311 auto analyzer using commercial kits from Roche Company (Roche Diagnostics, Mannheim, Germany).

Study cases were selected randomly from the STEPS, and then each case was then matched to control according to age, sex, and BMI. All patients were diagnosed with diabetes according to the diabetes diagnosis and treatment standards of the American Diabetes Association (ADA)^42^. The study protocol was approved by the ethics committee of Endocrinology and Metabolism Clinical Sciences Institute, Tehran University of Medical Sciences (IR.TUMS.EMRI.REC. 1395.00141) and performed under the declaration of Helsinki. Also, written informed consent was obtained from all participants.

### Detection of metabolites by tandem mass spectrometry

Details of the analytical procedures were described elsewhere^43^. In brief, fasting plasma samples were derivatized (with 1-butanol and HCL) and analyzed by the API SCIEX 3200 triple quadrupole mass spectrometer system, along with Thermo Scientific Dionex UltiMate 3000 standard HPLC system using positive electrospray ionization mode. A mixture of 75% acetonitrile aqueous solution was used as the mobile phase. The injected volume was five μL. The complete analysis contained 50 metabolites, including 20 amino acids and 30 acylcarnitines. The quantification of different metabolites was undertaken by Multiquant software (AB Sciex) against different isotopes as internal standards. Quality control was accomplished to screen the stability and functionality of the system through instrumental analyses.

### Metabolomics study

#### Data pre-processing

All data involved in this study were analyzed by IBM SPSS Statistics software version 26 (https://www.ibm.com/analytics/spss-statistics-software). Missing values were replaced based on imputation models and data scaled and standardized by Z-transformation to compare values between different data ranges.

#### Univariate analysis

The results were compared using independent sample T-test or Mann–Whitney U test (after checking the normality by Kolmogorov–Smirnov test), and *P* values were calculated. The odds ratio (OR) with a 95% confidence interval (CI) (per metabolite) was calculated using binary logistic regression analysis to adjust the results for systolic and diastolic blood pressure and lipid profile (HDL-C, cholesterol, triglyceride) as covariates. The Benjamini–Hochberg false discovery rate (FDR) was calculated to adjust the *P* value for multiple comparisons. *P* values < 0.05 were considered significant. Also, metabolites differences among diabetes and non-diabetes were evaluated according to sex differences.

#### Multivariate data analysis

The correlations of metabolites were calculated based on Pearson correlation coefficient (Supplementary Fig. 2), and then to deal with multiple comparisons, factor analysis was used, and principal component analysis (PCA) was performed by varimax rotation to extract factors, achieve the metabolite patterns and loading matrix. Kaiser–Meyer–Olkin (KMO) and Bartlett sphericity tests were used to evaluate the statistical correlation between variables and sufficiency of sample size. A KMO coefficient around 0.8 took into account as credible. We extracted 13 factors based on scree plot (eigenvalue > 1). Metabolites with the maximum loading (more than 0.4) for a factor were used as relevant components. Factor scores for each of the extracted factors were calculated by summing the concentrations of the metabolites multiplied by loadings. Multivariate logistic regression was used to estimate OR and their 95% CI of the extracted factors to analyze the relationship between metabolite patterns and diabetes incidence. Models were adjusted for blood pressure and lipid profile (HDL-C, cholesterol, triglyceride).

#### Pathways analyses

Influenced pathways for significant metabolites were plotted. Metaboanalyst software version 5.0 (https://www.metaboanalyst.ca )was implemented for pathway enrichment analysis based on the Kyoto Encyclopedia of Genes and Genomes (KEGG) pathway database.

### Acylcarnitines name

Free carnitine (C0), acetylcarnitine (C2), propionylcarnitine (C3), Malonylcarnitine (C3-DC), butyrylcarnitine (C4), Methylmalonyl-/succinylcarnitine (C4-DC), 3-OH-iso-/butyrylcarnitine (C4-OH), isovalerylcarnitine (C5), Tiglylcarnitine (C5:1), 3-OH-isovalerylcarnitine (C5-OH), glutarylcarnitine (C5DC), hexanoylcarnitine (C6), octanoylcarnitine (C8), Octenoylcarnitine (C8:1), decanoylcarnitine (C10), Decenoylcarnitine (C10:1), dodecanoylcarnitine (C12), tetradecanoylcarnitine (C14), Tetradecenoylcarnitine (C14:1), Tetradecadienoylcarnitine (C14:2), 3-OH-tetradecanoylcarnitine (C14-OH), hexadecanoylcarnitine (C16), 3-OH-hexadecanoylcarnitine (C16-OH), 3-OH-hexadecenoylcarnitine (C16:1-OH), Hexadecenoylcarnitine (C16:1), octadecanoylcarnitine (C18), Octadecenoylcarnitine (C18:1), 3-OH-octadecanoylcarnitine (C18-OH), 3-OH-octadecenoylcarnitine (C18:1-OH), Octadecadienoylcarnitine (C18:2).

## Supplementary Information

Supplementary Information.

## Data Availability

The datasets generated during and/or analyzed during the current study are available from the corresponding author on reasonable request.

## Abbreviations

NCD: Non-communicable disease
BMI: Body mass index
FPG: Fasting plasma glucose
HDL-C: High-density lipoprotein cholesterol
ADA: American Diabetes Association
OR: Odds ratio
CI: Confidence interval
KEGG: Kyoto Encyclopedia of Genes and Genomes
FDR: False discovery rate
PCA: Principal component analysis
KMO: Kaiser–Meyer–Olkin
BCAA: Branched-chain amino acids
AAA: Aromatic amino acids
T2D: Type 2 diabetes
T1D: Type 1 diabetes
TCA: Tricarboxylic acid
