# Supplementary material for: Targeted metabolomics analysis of amino acids and acylcarnitines as risk markers for diabetes by LC–MS/MS technique
Source: Sci Rep. 2022 May 19;12:8418. doi: 10.1038/s41598-022-11970-7 (PMC9119932; doi:10.1038/s41598-022-11970-7)
Supplement: Supplementary file 1 — Supplementary Information. [file 41598_2022_11970_MOESM1_ESM.docx]

**Targeted Metabolomics Analysis of Amino Acids and Acylcarnitines as Risk Markers for Diabetes by LC-MS/MS Technique**

Shaghayegh Hosseinkhani^1,2^, Babak Arjmand^3^, Arezou Dilmaghani-Marand^4^, Sahar Mohammadi Fateh^4^, Hojat Dehghanbanadaki^5^, Niloufar Najjar^6^, Sepideh Alavi-Moghadam^6^, Robabeh Ghodssi-Ghassemabadi^7^, Ensieh Nasli-Esfahani^1^, Farshad Farzadfar^4,8^, Bagher Larijani^8^, Farideh Razi^6,1^*

**Supplementary Table 1.** Significantly altered metabolites among groups’ classification using independent sample T-test or Mann-Whitney U test.

| **No.** | **Metabolites (µmol/L)** | **Non-diabetes** | **Diabetes** | **P-value** | **FDR** |
| --- | --- | --- | --- | --- | --- |
| 1 | C2 | 14.621±0.297 | 15.527±0.307 | **0.020** | **0.048** |
| 2 | C3 | 0.881±0.0263 | 0.955±0.025 | **0.009** | **0.028** |
| 3 | C3DC | 0.082±0.003 | 0.102±0.004 | **0.000** | **0.000** |
| 4 | C4 | 0.4816±0.027 | 0.527±0.03 | **0.034** | 0.076 |
| 5 | C4OH | 0.055±0.002 | 0.069±0.002 | **0.000** | **0.000** |
| 6 | C4DC | 0.07±0.002 | 0.102±0.004 | **0.000** | **0.000** |
| 7 | C5 | 0.230±0.007 | 0.245±0.007 | **0.046** | 0.090 |
| 8 | C5:1 | 0.04±0.002 | 0.058±0.003 | **0.000** | **0.000** |
| 9 | C5OH | 0.064±0.001 | 0.073±0.001 | **0.000** | **0.000** |
| 10 | C14OH | 0.013±0.000 | 0.015±0.001 | **0.004** | **0.013** |
| 11 | C16OH | 0.012±0.000 | 0.013±0.001 | **0.001** | **0.004** |
| 12 | C18 | 0.071±0.002 | 0.066±0.001 | **0.037** | 0.077 |
| 13 | C18:1 | 0.194±0.005 | 0.180±0.005 | **0.017** | **0.045** |
| 14 | C18OH | 0.009±0.000 | 0.011±0.000 | **0.000** | **0.000** |
| 15 | C18:2OH | 0.030±0.001 | 0.035±0.002 | **0.035** | 0.076 |
| 16 | Alanine | 417.00±6.146 | 468.514±6.146 | **0.000** | **0.000** |
| 17 | Leucine | 121.671±1.718 | 138.766±2.052 | **0.000** | **0.000** |
| 18 | Valine | 255.130±3.388 | 292.508±4.084 | **0.000** | **0.000** |
| 19 | Arginine | 70.627±1.364 | 66.965±1.316 | **0.020** | **0.048** |
| 20 | Citrulline | 39.494±0.710 | 36.896±0.813 | **0.017** | **0.045** |
| 21 | Glycine | 269.422±5.398 | 246.747±5.196 | **0.001** | **0.004** |
| 22 | Ornithine | 94.047±1.834 | 86.963±1.433 | **0.011** | **0.032** |
| 23 | Threonine | 139.347±2.268 | 128.614±2.406 | **0.000** | **0.000** |
| 24 | Serine | 102.895±1.962 | 91.101±1.825 | **0.000** | **0.000** |
| 25 | Histidine | 82.907±1.0814 | 77.1647±1.257 | **0.001** | **0.004** |
| 26 | Tryptophan | 69.722±1.0317 | 67.251±1.324 | **0.047** | 0.090 |
|  | Concentrations are reported as mean± SEM. | |  |  |  |

**Supplementary Table 2.** The odds ratio (OR) with a 95% confidence interval (CI) (per metabolite) was calculated using binary logistic regression analysis.

| **Metabolites** | **OR** | **95% CI** | | | **P- value** | **P- value †** | **P- value †** |
| --- | --- | --- | --- | --- | --- | --- | --- |
| C0 | 0.904 | (0.744 | ­- | 1.098) | 0.484 | 0.516 | 0.196 |
| C2 | 1.236 | (1.014 | ­- | 1.508) | 0.095 | 0.095 | 0.167 |
| C3 | 1.217 | (0.998 | ­- | 1.485) | 0.115 | 0.146 | 0.814 |
| C3DC | 1.620 | (1.250 | ­- | 2.098) | **0.000** | **0.000** | **0.000** |
| C4 | 1.128 | (0.912 | ­- | 1.395) | 0.435 | 0.383 | 0.517 |
| C4OH | 1.870 | (1.442 | ­- | 2.425) | **0.000** | **0.000** | **0.000** |
| C4DC | 3.009 | (2.159 | ­- | 4.194) | **0.000** | **0.000** | **0.000** |
| C5 | 1.155 | (0.948 | ­- | 1.406) | 0.273 | 0.304 | 0.448 |
| C5:1 | 2.013 | (1.509 | ­- | 2.687) | **0.000** | **0.000** | **0.000** |
| C5OH | 1.600 | (1.277 | ­- | 2.005) | **0.000** | **0.000** | **0.000** |
| C5DC | 1.033 | (0.852 | ­- | 1.254) | 0.885 | 0.906 | 0.814 |
| C6 | 1.084 | (0.892 | ­- | 1.318) | 0.576 | 0.592 | 0.694 |
| C8 | 1.021 | (0.841 | ­- | 1.239) | 0.885 | 0.906 | 0.900 |
| C8:1 | 1.087 | (0.895 | ­- | 1.320) | 0.576 | 0.559 | 0.446 |
| C10 | 0.977 | (0.805 | ­- | 1.186) | 0.885 | 0.906 | 1.000 |
| C10:1 | 0.974 | (0.803 | ­- | 1.182) | 0.885 | 0.906 | 1.000 |
| C12 | 0.969 | (0.798 | ­- | 1.177) | 0.885 | 0.756 | 1.000 |
| C14 | 0.977 | (0.804 | ­- | 1.187) | 0.885 | 0.798 | 1.000 |
| C14:1 | 0.923 | (0.758 | ­- | 1.123) | 0.576 | 0.531 | 0.900 |
| C14:2 | 0.930 | (0.766 | ­- | 1.130) | 0.597 | 0.592 | 1.000 |
| C14OH | 1.391 | (1.095 | ­- | 1.767) | **0.022** | **0.028** | 0.067 |
| C16 | 1.078 | (0.888 | ­- | 1.309) | 0.589 | 0.675 | 0.927 |
| C16OH | 1.470 | (1.158 | ­- | 1.866) | **0.008** | **0.000** | **0.036** |
| C16:1OH | 1.012 | (0.834 | ­- | 1.229) | 0.939 | 0.959 | 1.000 |
| C16:1 | 0.954 | (0.786 | ­- | 1.158) | 0.791 | 0.654 | 1.000 |
| C18 | 0.812 | (0.664 | ­- | 0.992) | 0.100 | 0.075 | 0.105 |
| C18:1 | 0.808 | (0.659 | ­- | 0.991) | 0.100 | 0.075 | 0.250 |
| C18OH | 1.397 | (1.102 | ­- | 1.771) | **0.020** | **0.028** | **0.036** |
| C18:1OH | 1.117 | (0.918 | ­- | 1.359) | 0.435 | 0.545 | 0.385 |
| C18:2OH | 1.360 | (1.070 | ­- | 1.729) | **0.035** | **0.028** | 0.088 |
| Alanine | 1.860 | (1.488 | ­- | 2.325) | **0.000** | **0.000** | **0.000** |
| Aspartic Acid | 1.006 | (0.829 | ­- | 1.221) | 0.952 | 0.980 | 1.000 |
| Glutamic Acid | 1.156 | (0.950 | ­- | 1.407) | 0.273 | 0.269 | 0.842 |
| Leucine | 2.010 | (1.585 | ­- | 2.549) | **0.000** | **0.000** | **0.000** |
| Methionine | 0.882 | (0.725 | ­- | 1.072) | 0.357 | 0.328 | 0.280 |
| Phenylalanine | 1.243 | (1.000 | ­- | 1.544) | 0.114 | 0.130 | 0.444 |
| Tyrosine | 1.082 | (0.891 | ­- | 1.314) | 0.576 | 0.592 | 0.838 |
| Valine | 2.197 | (1.717 | ­- | 2.812) | **0.000** | **0.000** | **0.000** |
| Arginine | 0.825 | (0.678 | ­- | 1.004) | 0.115 | 0.130 | 0.105 |
| Citrulline | 0.785 | (0.643 | ­- | 0.959) | **0.048** | **0.028** | 0.125 |
| Glycine | 0.738 | (0.603 | ­- | 0.902) | **0.011** | **0.000** | **0.036** |
| Ornithine | 0.732 | (0.595 | ­- | 0.900) | **0.011** | **0.000** | 0.088 |
| Proline | 1.172 | (0.961 | ­- | 1.431) | 0.236 | 0.240 | 0.814 |
| Threonine | 0.721 | (0.589 | ­- | 0.883) | **0.008** | **0.000** | **0.000** |
| Serine | 0.626 | (0.502 | ­- | 0.781) | **0.000** | **0.000** | **0.000** |
| Histidine | 0.706 | (0.577 | ­- | 0.864) | **0.005** | **0.000** | **0.000** |
| Lysine | 1.029 | (0.848 | ­- | 1.249) | 0.885 | 0.906 | 0.814 |
| Tryptophan | 0.863 | (0.709 | ­- | 1.051) | 0.273 | 0.304 | 0.182 |
| Asparagine | 0.920 | (0.758 | ­- | 1.117) | 0.576 | 0.592 | 0.838 |
| Glutamine | 0.991 | (0.817 | ­- | 1.203) | 0.947 | 0.906 | 1.000 |

P-value, crude model

P-value†, adjusted by blood pressure

P-value††, adjusted by blood pressure and lipid profile (HDL-C, cholesterol, triglyceride)

OR has been reported for the crude model.

All P-values are adjusted for multiple testing using the Benjamin-Hochberg false discovery rate.

**Supplementary Table 3.** Significantly altered metabolites among groups’ classification using independent sample T-test or Mann-Whitney U test according to sex differences.

|  | **Non-diabetes** | | | | | **Diabetes** | | | | |
| --- | --- | --- | --- | --- | --- | --- | --- | --- | --- | --- |
| **Metabolites (µmol/L)** | **Female** | | **Male** | | **P-value** | **Female** | | **Male** | | **P-value** |
|  | mean | SEM | mean | SEM |  | mean | SEM | mean | SEM |  |
| C0 | 59.191 | 1.047 | 56.312 | 1.355 | 0.238 | 55.688 | 1.220 | 57.700 | 1.463 | 0.676 |
| C2 | 15.057 | 0.386 | 14.092 | 0.459 | 0.171 | 15.843 | 0.395 | 15.150 | 0.480 | 0.300 |
| C3 | 0.844 | 0.030 | 0.932 | 0.045 | 0.360 | 0.880 | 0.031 | 1.044 | 0.039 | **0.008** |
| C3DC | 0.081 | 0.003 | 0.084 | 0.004 | 0.600 | 0.097 | 0.006 | 0.108 | 0.006 | 0.086 |
| C4 | 0.462 | 0.019 | 0.504 | 0.056 | 0.430 | 0.527 | 0.040 | 0.528 | 0.045 | 0.963 |
| C4OH | 0.055 | 0.002 | 0.055 | 0.004 | 0.238 | 0.071 | 0.003 | 0.066 | 0.002 | 0.580 |
| C4DC | 0.070 | 0.002 | 0.070 | 0.002 | 0.799 | 0.100 | 0.005 | 0.104 | 0.006 | 0.826 |
| C5 | 0.219 | 0.010 | 0.245 | 0.010 | **0.025** | 0.226 | 0.009 | 0.266 | 0.010 | **0.008** |
| C5:1 | 0.043 | 0.003 | 0.036 | 0.002 | 0.519 | 0.057 | 0.004 | 0.059 | 0.004 | 0.676 |
| C5OH | 0.064 | 0.002 | 0.064 | 0.002 | 0.907 | 0.071 | 0.002 | 0.075 | 0.002 | 0.083 |
| C5DC | 0.308 | 0.011 | 0.350 | 0.011 | **0.008** | 0.310 | 0.010 | 0.355 | 0.011 | **0.008** |
| C6 | 0.218 | 0.016 | 0.177 | 0.008 | 0.579 | 0.217 | 0.014 | 0.202 | 0.011 | 0.852 |
| C8 | 0.328 | 0.027 | 0.310 | 0.021 | 0.626 | 0.326 | 0.026 | 0.324 | 0.023 | 0.691 |
| C8:1 | 0.372 | 0.018 | 0.312 | 0.016 | **0.025** | 0.402 | 0.021 | 0.310 | 0.015 | **0.017** |
| C10 | 0.434 | 0.036 | 0.418 | 0.028 | 0.547 | 0.417 | 0.031 | 0.421 | 0.029 | 0.676 |
| C10:1 | 0.398 | 0.027 | 0.379 | 0.023 | 0.922 | 0.375 | 0.024 | 0.393 | 0.023 | 0.548 |
| C12 | 0.149 | 0.010 | 0.155 | 0.007 | 0.276 | 0.146 | 0.007 | 0.152 | 0.007 | 0.620 |
| C14 | 0.061 | 0.004 | 0.060 | 0.002 | 0.547 | 0.059 | 0.002 | 0.061 | 0.002 | 0.472 |
| C14:1 | 0.141 | 0.009 | 0.131 | 0.007 | 0.579 | 0.129 | 0.006 | 0.132 | 0.006 | 0.821 |
| C14:2 | 0.100 | 0.005 | 0.100 | 0.005 | 0.842 | 0.091 | 0.004 | 0.102 | 0.004 | 0.077 |
| C14OH | 0.013 | 0.001 | 0.013 | 0.001 | 0.922 | 0.015 | 0.001 | 0.016 | 0.001 | 0.557 |
| C16 | 0.184 | 0.005 | 0.190 | 0.006 | 0.579 | 0.189 | 0.005 | 0.193 | 0.006 | 0.965 |
| C16OH | 0.011 | 0.000 | 0.012 | 0.000 | 0.519 | 0.013 | 0.001 | 0.014 | 0.001 | 0.608 |
| C16:1OH | 0.019 | 0.001 | 0.018 | 0.001 | 0.922 | 0.019 | 0.001 | 0.020 | 0.001 | 0.689 |
| C16:1 | 0.054 | 0.002 | 0.046 | 0.002 | 0.050 | 0.051 | 0.002 | 0.048 | 0.002 | 0.261 |
| C18 | 0.068 | 0.002 | 0.074 | 0.003 | 0.108 | 0.064 | 0.002 | 0.068 | 0.002 | 0.437 |
| C18:1 | 0.200 | 0.008 | 0.188 | 0.006 | 0.576 | 0.176 | 0.006 | 0.185 | 0.007 | 0.691 |
| C18OH | 0.009 | 0.000 | 0.009 | 0.001 | 0.547 | 0.011 | 0.001 | 0.011 | 0.001 | 0.938 |
| C18:1OH | 0.013 | 0.001 | 0.013 | 0.001 | 0.519 | 0.014 | 0.001 | 0.014 | 0.001 | 0.691 |
| C18:2OH | 0.032 | 0.001 | 0.028 | 0.001 | 0.467 | 0.035 | 0.002 | 0.035 | 0.002 | 0.691 |
| Alanine | 420.222 | 7.737 | 413.087 | 9.877 | 0.843 | 480.571 | 8.028 | 454.148 | 9.313 | 0.082 |
| Aspartic Acid | 12.616 | 0.390 | 12.861 | 0.366 | 0.448 | 12.200 | 0.354 | 13.405 | 0.402 | 0.082 |
| Glutamic Acid | 64.985 | 1.100 | 70.794 | 1.317 | **0.008** | 68.585 | 1.362 | 70.528 | 1.270 | 0.234 |
| Leucine | 115.147 | 2.077 | 129.599 | 2.634 | **0.000** | 132.503 | 2.580 | 146.228 | 3.126 | **0.008** |
| Methionine | 27.920 | 0.574 | 29.365 | 0.497 | **0.008** | 26.513 | 0.477 | 29.469 | 0.647 | **0.000** |
| Phenylalanine | 63.715 | 1.192 | 65.368 | 1.200 | 0.276 | 66.494 | 1.898 | 68.387 | 1.157 | **0.030** |
| Tyrosine | 72.534 | 1.561 | 73.955 | 1.476 | 0.430 | 72.239 | 1.355 | 76.942 | 1.655 | 0.082 |
| Valine | 247.912 | 4.585 | 263.901 | 4.903 | **0.025** | 285.283 | 5.350 | 301.118 | 6.196 | 0.134 |
| Arginine | 69.852 | 1.727 | 71.569 | 2.179 | 0.547 | 64.104 | 1.549 | 70.374 | 2.174 | 0.086 |
| Citrulline | 38.435 | 0.983 | 40.781 | 1.013 | 0.100 | 34.881 | 1.089 | 39.297 | 1.179 | **0.017** |
| Glycine | 292.531 | 8.192 | 241.344 | 5.380 | **0.000** | 264.802 | 7.942 | 225.235 | 5.617 | **0.008** |
| Ornithine | 93.025 | 2.319 | 95.289 | 2.936 | 0.807 | 84.470 | 1.865 | 89.933 | 2.191 | 0.145 |
| Proline | 242.242 | 7.686 | 275.344 | 7.840 | **0.000** | 265.862 | 9.107 | 275.825 | 8.201 | 0.300 |
| Threonine | 137.340 | 2.952 | 141.785 | 3.520 | 0.519 | 124.729 | 3.307 | 133.243 | 3.461 | 0.086 |
| Serine | 107.263 | 2.939 | 97.587 | 2.378 | 0.247 | 91.210 | 2.688 | 90.971 | 2.411 | 0.900 |
| Histidine | 82.561 | 1.416 | 83.327 | 1.673 | 0.746 | 77.387 | 1.852 | 76.899 | 1.659 | 0.723 |
| Lysine | 179.782 | 3.793 | 174.663 | 4.698 | 0.480 | 177.431 | 4.011 | 180.196 | 4.357 | 0.840 |
| Tryptophane | 66.985 | 1.274 | 73.048 | 1.620 | **0.021** | 64.967 | 1.988 | 69.973 | 1.642 | **0.017** |
| Asparagine | 44.296 | 1.579 | 45.393 | 1.739 | 0.671 | 41.761 | 1.977 | 45.082 | 1.881 | 0.234 |
| Glutamine | 519.510 | 10.829 | 505.365 | 13.059 | 0.547 | 504.861 | 11.124 | 520.691 | 11.500 | 0.676 |

Concentrations are reported as mean± SEM.

All P-values are adjusted for multiple testing using the Benjamin-Hochberg false discovery rate.

**Supplementary Figure 1.** Scree plot for exploratory factor analysis (IBM SPSS Statistics software version 26).


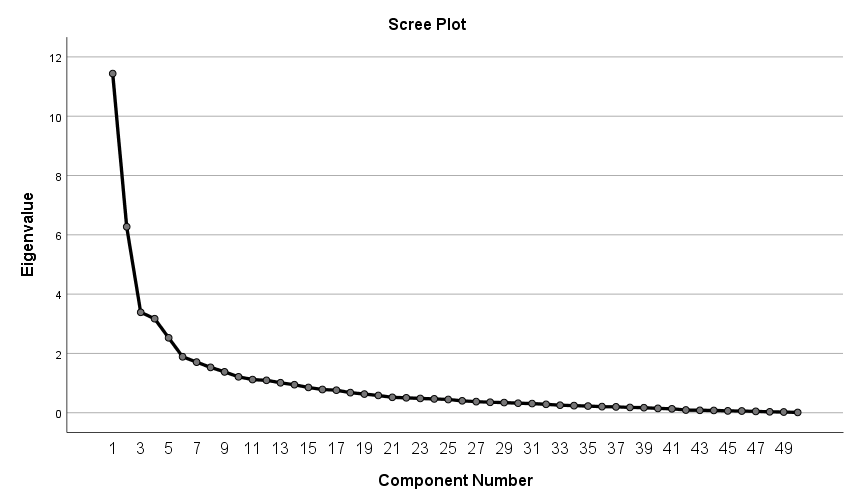


**Supplementary Figure 2.** Correlation heatmap based on Pearson correlation coefficient (Metaboanalyst software version 5.0).


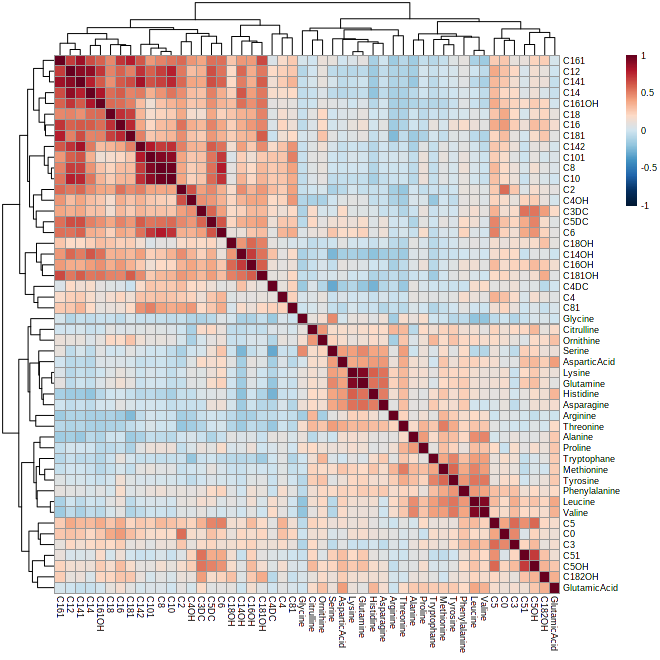


Metabolites with the most correlation coefficient:

Valine and leucine r = 0.923

Glutamine and lysine r = 0.922

C8 AND C10 r = 0.969

C8 AND C10:1 r = 0.924

C10 AND C10:1 r = 0.897

C12 AND C14 r = 0.885

C12 AND C14:1 r = 0.899

C14:1 AND C14:2 r = 0.83

**Supplementary Table 4.** Extracted factors based on PCA and loadings.

| **Factor 1** | **Loading** | **Factor 3** | **Loading** | **Factor 6** | **Loading** | **Factor 10** | **Loading** |
| --- | --- | --- | --- | --- | --- | --- | --- |
| C8 | 0.94 | Tyrosine | 0.794 | C16OH | 0.767 | Alanine | 0.719 |
| C10 | 0.936 | Leucine | 0.743 | C18OH | 0.749 | Proline | 0.719 |
| C10:1 | 0.935 | Valine | 0.723 | C14OH | 0.702 | **Factor 11** | **Loading** |
| C14:2 | 0.76 | Methionine | 0.721 | C18:1OH | 0.549 | C18:2OH | 0.711 |
| C6 | 0.742 | Tryptophan | 0.679 | **Factor 7** | **Loading** | Glutamic Acid | 0.588 |
| C14:1 | 0.672 | Phenylalanine | 0.604 | C3 | 0.714 | **Factor 12** | **Loading** |
| C12 | 0.659 | **Factor 4** | **Loading** | C0 | 0.685 | Citrulline | 0.818 |
| C5DC | 0.529 | Lysine | 0.865 | C4 | 0.528 | Ornithine | 0.591 |
| C14 | 0.399 | Glutamine | 0.848 | **Factor 8** | **Loading** | **Factor 13** | **Loading** |
| **Factor 2** | **Loading** | Asparagine | 0.785 | C4OH | 0.722 | Arginine | 0.83 |
| C16 | 0.831 | Histidine | 0.751 | C2 | 0.629 |  |  |
| C18:1 | 0.804 | Aspartic Acid | 0.544 | C8:1 | 0.62 |  |  |
| C18 | 0.796 | **Factor 5** | **Loading** | **Factor 9** | **Loading** |  |  |
| C16:1 | 0.71 | C5:1 | 0.886 | Serine | 0.674 |  |  |
| C14 | 0.688 | C5OH | 0.793 | Glycine | 0.63 |  |  |
| C16:1OH | 0.687 | C3DC | 0.675 | Threonine | 0.344 |  |  |
|  |  | C5 | 0.527 | C4DC | -0.624 |  |  |

**Supplementary Table 5.** Details of pathway analysis.

|  | **Metabolite Set** | **Total** | **Hits** | **Expect** | **P-value** | **FDR** |
| --- | --- | --- | --- | --- | --- | --- |
| ﻿ | Aminoacyl-tRNA biosynthesis | 48 | 6 | 0.347 | 2.94E-7 | 2.47E-5 |
| ﻿ | Valine, leucine and isoleucine biosynthesis | 8 | 3 | 0.0578 | 1.54E-5 | 6.49E-4 |
| ﻿ | Glycine, serine and threonine metabolism | 33 | 2 | 0.239 | 0.0222 | 0.621 |
| ﻿ | Valine, leucine and isoleucine degradation | 40 | 2 | 0.289 | 0.0319 | 0.67 |
| ﻿ | Histidine metabolism | 16 | 1 | 0.116 | 0.11 | 1.0 |
| ﻿ | Pantothenate and CoA biosynthesis | 19 | 1 | 0.137 | 0.129 | 1.0 |
| ﻿ | Selenocompound metabolism | 20 | 1 | 0.145 | 0.136 | 1.0 |
| ﻿ | beta-Alanine metabolism | 21 | 1 | 0.152 | 0.142 | 1.0 |
| ﻿ | Alanine, aspartate and glutamate metabolism | 28 | 1 | 0.202 | 0.185 | 1.0 |
| ﻿ | Glutathione metabolism | 28 | 1 | 0.202 | 0.185 | 1.0 |
| ﻿ | Porphyrin and chlorophyll metabolism | 30 | 1 | 0.217 | 0.197 | 1.0 |
| ﻿ | Glyoxylate and dicarboxylate metabolism | 32 | 1 | 0.231 | 0.209 | 1.0 |
| ﻿ | Primary bile acid biosynthesis | 46 | 1 | 0.332 | 0.287 | 1.0 |

Hit: actually matched number from the data

FDR: P-value adjusted using False Discovery Rate
